# Examining markers of safety in homecare using the international classification for patient safety

**DOI:** 10.1186/1472-6963-13-191

**Published:** 2013-05-24

**Authors:** Marilyn T Macdonald, Ariella Lang, Janet Storch, Lynn Stevenson, Tanya Barber, Kristine Iaboni, Susan Donaldson

**Affiliations:** 1 School of Nursing, Faculty of Health Professions, Dalhousie University, 5869 University Avenue, PO Box, 15000, Halifax, Nova Scotia, B3H 4R2, Canada; 2 VON Canada, 110 Argyle Avenue, Ottawa, Ontario, K2P 1B4, Canada; 3 School of Nursing, University of Victoria, STN CSC, PO Box 1700, Victoria, British Columbia, V8W 2Y2, Canada; 4 Vancouver Island Health Authority, 1952 Bay Street, Victoria, British Columbia, V8R 1J8, Canada; 5 School of Nursing, Faculty of Health Professions, Dalhousie University, 5869 University Avenue, PO Box, 15000, Halifax, Nova Scotia, B3H 4R2, Canada; 6 Department of Educational Psychology, Faculty of Graduate Studies, University of Alberta, 6-102 Education North Edmonton, Alberta, 11, T6G 2G5, Canada; 7 Canadian Home Care Association, 7111 Syntax Drive 3rd Floor, Mississauga, Ontario, LON 8C3, Canada

## Abstract

**Background:**

Homecare is a growth enterprise. The nature of the care provided in the home is growing in complexity. This growth has necessitated both examination and generation of evidence around patient safety in homecare. The purpose of this paper is to examine the findings of a recent scoping review of the homecare literature 2004-2011 using the World Health Organization International Classification for Patient Safety (ICPS), which was developed for use across all care settings, and discuss the utility of the ICPS in the home setting. The scoping review focused on Chronic Obstructive Pulmonary Disease (COPD), and Congestive Heart Failure (CHF); two chronic illnesses commonly managed at home and that represent frequent hospital readmissions. The scoping review identified seven safety markers for homecare: *Medication mania*; *Home alone*; *A fixed agenda in a foreign language*; *Strangers in the home*; *The butcher*, *the baker*, *the candlestick maker*; *Out of pocket*: *the cost of caring at home*; and *My health for yours*: *declining caregiver health*.

**Methods:**

The safety markers from the scoping review were mapped to the 10 ICPS high-level classes that comprise 48 concepts and address the continuum of health care: Incident Type, Patient Outcomes, Patient Characteristics, Incident Characteristics, Contributing Factors/Hazards, Organizational Outcomes, Detection, Mitigating Factors, Ameliorating Actions, and Actions Taken to Reduce Risk.

**Results:**

Safety markers identified in the scoping review of the homecare literature mapped to three of the ten ICPS classes: Incident Characteristics, Contributing Factors, and Patient Outcomes.

**Conclusion:**

The ICPS does have applicability to the homecare setting, however there were aspects of safety that were overlooked. A notable example is that the health of the caregiver is inextricably linked to the wellbeing of the patient within the homecare setting. The current concepts within the ICPS classes do not capture this, nor do they capture how care responsibilities are shared among patients, caregivers, and providers.

## Background

A steadily increasing demand for homecare services within Canada co-exists with the aging population. It is estimated that within the next 25 years seniors will make up 25% of the population [1]. In addition, The World Health Organization (WHO) 2010 Global Status report [2] indicated that developing one or more chronic illnesses is more likely with advancing age. The increasing reliance on homecare to provide health system services is therefore not surprising. What is surprising is the lack of focus on patient safety within the homecare context. We conducted a scoping review to begin to address this gap by identifying safety related markers for homecare patients, caregivers, and health care providers. A full report of the findings is published elsewhere (http://www.tandfonline.com/eprint/vxHsUwV68wbUXqKheD7p/full#.UaPkpkqmWIQ) [3]. The purpose of this paper is to employ the WHO International Classification for Patient Safety (ICPS) as a lens to re-present our findings. In particular we were interested in the applicability of the ICPS within the homecare context.

Research on patient safety has primarily stemmed from institutional settings. The Victorian Order of Nurses (VON), Canada’s largest not for profit homecare organization, approached the Canadian Patient Safety Institute (CPSI) in 2005 to address patient safety in homecare. Following a national roundtable discussion with researchers and decision makers, CPSI published *Broadening the Patient Safety Agenda to Include Homecare*[4]. This foundational report identified that the context of care in the home is different than in hospitals or institutional settings and that the health of the homecare patient and caregiver is inextricably linked. In addition, this report confirmed the lack of literature focusing on homecare safety. Subsequently, in 2008, the CPSI formed the Core Safety in Homecare Team with researchers and decision makers to further patient safety research in homecare [5-7]. Included in this program of research was a scoping review of the homecare literature to identify safety related markers for patients, their (unpaid/family) caregivers, and paid health providers. It should be noted that although it is customary within homecare research to use the term clients for those receiving care, we will use the WHO designated term, thus referring to clients as patients. The review focused on the homecare literature surrounding chronic obstructive pulmonary disease (COPD) and congestive heart failure (CHF). These two chronic illnesses require a great deal of care, most of which is provided in the home by a family caregiver [8], and have the highest readmission rate to acute care of all medical conditions [9]. In light of the accumulating evidence related to safety in homecare and the absence of literature linking safety in homecare with the ICPS, this manuscript was conceived.

In 2002, the WHO was urged to focus on patient safety and quality of care by developing global norms and standards, as well as supporting efforts to develop patient safety policies and practices. In order to meet this call, the WHO created the World Alliance for Patient Safety in October, 2004. One of its initiatives was to develop an International Classification for Patient Safety (ICPS), and a Drafting Group was formed to meet this initiative [10]. The Drafting Group’s main objective was to “define, harmonize and group patient safety concepts into an internationally agreed classification in a way that is conducive to learning and improving patient safety across systems” [10], p3. In order to meet this objective, the Group followed a set of principles that included developing “categories applicable to the full spectrum of health care settings” [10], p5 and required that that the framework be “a genuine convergence of international perceptions of the main issues related to patient safety” [10], p6. The framework consists of 10 high level classes: 1) Incident Type; 2) Patient Outcomes; 3) Patient Characteristics; 4) Incident Characteristics; 5) Contributing Factors/Hazards; 6) Organizational Outcomes; 7) Detection; 8) Mitigating Factors; 9) Ameliorating Actions; and 10) Actions Taken to Reduce Risk. The framework also consists of a number of associated concepts, and is not considered a comprehensive classification of patient safety [10].

The conceptual framework “was designed to provide a much needed model for organizing patient safety data and information so that it can be aggregated and analyzed to compare patient safety data across disciplines, between organizations, and across time and borders; examine the roles of system and human factors in patient safety; identify potential patient safety issues; and develop priorities and safety solutions” [10], p4. In consideration of its goals, purposes, and principles, we questioned if the ICPS framework was applicable to patient safety within the homecare context. While studies exist demonstrating its successful application to research findings [11,12], there are also those that have critiqued the ICPS and its applicability as a classification system [13]. In all these cases, the ICPS was evaluated through and utilized in clinical institutional settings and contexts. In order to verify this classification system and its potential to apply to “the full spectrum of health care settings” we have used the ICPS as a lens with which to present our scoping review findings.

In this paper we view the results of our scoping review, which identified markers that may compromise the safety of homecare patients and their unpaid caregivers, through the lens of the ICPS. The markers are introduced and explained in the process of examining them through the high classes of the ICPS framework.

## Methods

The purpose of a scoping review is to allow the researcher to explore the breadth of a topic, in our case, the home care literature related to COPD and CHF. All research designs are included in the review. Unlike a systematic review, a scoping review does not begin with a defined question [14]. We used the following methods in order to assure trustworthy review findings: describing the sources of the data; developing inclusion/exclusion criteria; searching relevant work based on predetermined criteria; collecting the data; and synthesizing the data and explaining the method for synthesis. The sources of data searched included 12 electronic bibliographic databases and five sources of grey literature. The inclusion/exclusion criteria considered: geographical location of study (North America, European Union, Australia), year of publication (2004-2011), and language (French and English). The predetermined search criteria included the terms COPD and CHF in the title fields, as well as a developed list of search terms. This resulted in 376 articles. Further screening involving a three stage triaging process where selected team members ranked the titles, abstracts, or full-text articles as relevant, potentially relevant, and not relevant, resulted in 180 relevant articles. These articles were analyzed by both researcher and decision-maker team members using interpretive description methods [15] to identify patterns that would represent safety-related markers for patients and their unpaid caregivers.

## Results

### Applying the 10 high-level classes to findings

The ten high-level classes outlined above have been grouped to enhance meaning. One grouping is Incident Type and Patient Outcomes. The second grouping is context relevant and contains the classes of Patient Characteristics, Incident Characteristics, and Contributing Factors. A third grouping captures information related to prevention, recovery, and system resilience and is comprised of Detection, Mitigating Factors, Ameliorating Actions and Actions Taken to Reduce Risk. The safety markers identified in the scoping review map to the high-level classes as follows: *Medication mania* fits in Incident Characteristics; *Home alone*, *A fixed agenda in a foreign language*, *Strangers in the home*, and *The butcher*, *the baker*, *the candlestick maker* are Contributing Factors; and *Out of pocket*: *the cost of caring at home* and *My health for yours*: *declining caregiver health* are Patient Outcomes.

**Figure 1:**
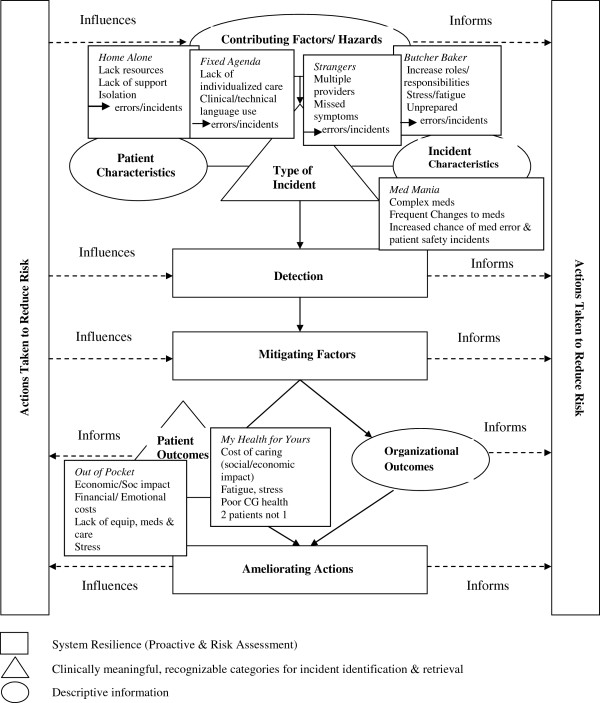
**The conceptual framework for the international classification for patient safety [**[10]**], p.8.** ©WHO, 2009. All Rights Reserved. WHO/IER/PSP/2010.2. Permission obtained for reproduction. The seven safety markers that resulted from our Scoping Review: Medication mania; Home alone; A fixed agenda in a foreign language; Strangers in the home; The butcher, the baker, the candlestick maker; Out of pocket: the cost of caring at home; and My health for yours: declining caregiver health were mapped to the high-level classes of the ICPS. The findings mapped to three of the high-level classes: Contributing Factors/Hazards; Incident Characteristics; and Patient Outcomes as shown.

The high-level classes and the safety markers will now be defined, and the markers presented in relation to their fit with the respective high-level classes and associated concepts. The safety marker definitions were derived from the data in the scoping review and used to map the markers to the framework high classes (see Figure 1).

### Incident characteristics

The class Incident Characteristics is defined in the ICPS as *classifying the information about the circumstances surrounding the incident such as where and when the incident occurred*, *who was involved*, *and who reported the incident*. The three main concepts under this class include Origin of Incident, Discovery of Incident, and Reporting of Incident. *Medication mania* is a safety marker identified through the scoping review that refers to the number and complexity of medications patients are required to take and manage. In viewing this safety marker through the lens of the high-level class Incident Characteristics, it was apparent that the setting of homecare and the role of the unpaid caregivers were recognized. However, we also discovered that while a link existed within this class between the patient and caregiver, the dynamic or importance of the relationship between patient and caregiver was not clearly depicted by the framework.

For instance, this marker can map to the concept of Origin of Incident in considering the complex nature of medication regimes for individuals with chronic illness and the risk of medication errors. Patients with severe heart failure may follow medication regimes that include five or more medications while those with co-morbid conditions may follow regimes with 10 or more medications [16]. For both caregivers and patients, having to monitor such complex regimes may lead to safety concerns such as duplicating, omitting, or misusing medications [17-20]; forgetting or confusing the number of medications to take, when to take, and which medications to avoid mixing [21-23]; medication errors [24,25]; worsening of symptoms and increased exacerbations [24]; higher risk of anxiety and potential overdose [24-28]; and health decline [16,22,25].

These medication errors can be linked to caregivers, either because they manage the regime, or they discover or report the incident. However, the framework does not depict how or why caregivers can be linked to patient medication errors. The framework seems to focus on the care setting and timing of the incident rather than to consider the dynamic and complex relationship that exists between patient and caregiver, health, and care related incidents.

For example, while the Origin of Incident includes the sub-concepts of

‘Who’, listing those who may be involved, including caregivers, and ‘When’ or Stage of Care, listing Homecare and Management of Household Regime, it does not go further to consider the characteristics of the caregiver and the caregiver’s health. These are key considerations because the age and health of the caregiver will determine if he/she also has a medication regime to manage on top of assisting with the patient’s [18,29]. The literature shows that caregivers may experience increased stress when having to monitor both their own medications and that of the homecare patient [18,29]. Additionally, caregivers are more likely to make mistakes as their own health declines under the pressures and burden of their rising care responsibilities [21,30]. Caregivers in the literature reported feeling the expectation to take on multiple responsibilities with little information or preparation regarding how to manage their loved one’s illness [31].

It is evident that while we can view our findings relating to the marker *Medication mania* through the concepts that exist under Incident Characteristics, the framework relegates caregivers to the role of discovery or reporting of safety-related incidents, thereby missing the true nature of the caregiver’s role in homecare settings and their relationship to the patient and the patient’s health.

### Contributing factors/hazards

This class is defined within the ICPS as *circumstances*, *actions*, *or influences which are thought to have played a part in the origin or development of an incident or to increase risk of an incident*. We mapped four safety markers to the high-level class Contributing Factors: *Home alone*; *A fixed agenda in a foreign language*; *Strangers in the home*; and *The butcher*, *the baker*, *the candlestick maker*. *Home alone* is defined in the scoping review as patients feeling left on their own to deal with their illness without adequate information or support. *A fixed agenda in a foreign language* is defined as the perception that information offered by health care providers regarding illness often follows a fixed script and uses medical or clinical terminology that can be hard to understand, sounding like a foreign language to patients and caregivers. *Strangers in the home* is defined as the interaction between homecare patients and the multiple and various health professionals coming into their homes to provide care. And *The butcher*, *the baker*, *the candlestick maker* describes the increasing responsibilities and multiple roles caregivers take on, thus becoming the butcher, the baker, the candlestick maker, and any other role necessary in caring for loved ones.

Many of the concepts associated with Contributing Factors related well to our findings. However, the unique setting of receiving care at home was not accounted for, as we will illustrate. Staff Factors, a concept of Contributing Factors, fit with our findings relating to *Home alone*, *A fixed agenda* and *Strangers in the home*. In these three markers we identified staff communication as a key issue. For example, the literature revealed that patients and caregivers perceived health professionals as lacking time to answer questions, provide clear information, and grasp their individual needs [32,33]. Patients and caregivers also described information, education, or communication provided by health care professionals as insufficient or inadequate, which lead to feelings of neglect, abandonment, and being left on their own to deal with their illness [31,32,34].

In addition, health professionals were seen to provide sporadic information [35,36], use language that was too technical or clinical thus limiting patient and caregiver’s ability to be involved in their own care [37], use euphemisms that weakened understandings of the health issues at hand [38], and provide conflicting information and approaches regarding treatments [38]. This lack of specific information or failure to explain information in clear terms was linked to greater patient anxiety as well as strain on caregivers, leading to mismanagement of symptoms and thus increased patient hospitalizations [32,34,39].

Staff Factors described in the ICPS, such as the sub-concepts of Performance Factors and Behaviour, overlook key staff issues in homecare, such as collaboration and coordination. If providers do not collaborate or coordinate amongst themselves as well as with patients and caregivers, this can negatively impact on patients’ and caregivers’ lives. For instance, patients and caregivers must often work with numerous health professionals from multiple independent agencies [38]. As a result, they may feel overwhelmed, insecure, and uncomfortable with the number of ‘strangers’ in their home, leading to feelings of confusion or being a burden [39,40]. This can, in turn, result in a further decline in overall collaboration among health professionals and patients [39-43], and can contribute to the possibility of: care errors and poor health [30,31,44]; patient stress and fatigue [31,44]; feelings of anxiety and depression from loss of control within the home [36]; and re-hospitalization due to care errors such as the omission or duplication of tasks [31,44].

Patient Factors, another concept within Contributing Factors, is also useful in presenting our findings as the sub-concepts Cognitive, Performance, Behaviour, Communication, Emotional, and Social factors can easily be applied to the four identified markers. For example, the literature shows that aspects associated with patients include difficulties in the reception and comprehension of information due to cognitive impairments such as memory loss or decreased functionality due to illness or medications [45-49]. Challenges with technology also occur as the symptoms of illness, such as diminished attention and slower motor reactions in patients with CHF, make it more difficult for patients to learn to use equipment or devices [19,45,50-52].

Also within Contributing Factors, Emotional, Behavioural, and Social Factors were seen as applicable concepts to our findings. This is illustrated in that a diagnosis of COPD or CHF was strongly linked to: feelings of isolation, depression, anxiety, decreased quality of life, and being housebound or left alone due to the associated symptoms of the illness (breathlessness, fatigue, insomnia, headaches, falls and reduced mobility); unpredictability of disease process and progression; and diminished physical ability, which limits a patient’s ability to participate in activities and social engagements [19,31,34,40,53-56]. Patients also resisted asking for help, which increased feelings of isolation, due to shame, guilt, and embarrassment, or not wanting to be a burden to family or friends [22,31,32]. Cultural and language barriers were also identified as issues whereby the homecare team (patient, caregiver, and health professionals) may not be communicating and understanding each other’s perceptions of health and healing [57].

Despite three of the four markers mapping quite smoothly to the high-level class of Contributing Factors, our third marker, *Butcher*, *baker*, *candlestick maker*, illustrates a weakness in sensitivity of the ICPS framework to the homecare context. Caregivers’ behaviour, performance, cognitive, communication, emotional, and social factors have a significant impact on how the client is cared for at home, and thus should be considered as concepts within this class. Caregivers must take on multiple roles [58,59]; experience changes in family dynamics and boundaries [60,61]; and face an increasing work load in caring for a loved one (see Table 1 for a list of responsibilities described in the literature that caregivers are expected to take on) on top of their existing responsibilities and employment [31,59,62,63]. Assuming these roles and responsibilities can lead to frustration, resentment, anxiety, stress, exhaustion, and declining health [30,35,64-66]. Consequently, patients may be at risk for medical and care errors as their caregiver’s health declines, or abuse from caregivers who feel frustrated or resentful due to increasing demands and stress [21,30,67].

**Table 1:** Caregiver responsibilities

	
• Medication and symptom management [17,59,61,64,68-71]	• Physical work (e.g., moving a frail client, bathing, laundry and cleaning, etc.) [18,39,69]
• Awareness of hygiene and nutritional needs and exercise regimes [17,32,69,72-74]	• Use of technical devices and equipment (e.g., defibrillators, IVs, ventricular assistant devices, insulin pens, home dialysis devices, etc.) [17,71,74,79,80]
• Providing physical, psychological and emotional support (e.g., transportation, company or social support) [42,69,74-76].	
	• Managing client behaviours and temperaments [18,29,75,81]
• Emergency management of issues (e.g., trouble breathing, pain management, recognizing signs and symptoms of problems) [18,69,71,77,78]	
	• Handling household and illness related finances [59,68,75]
	• Carrying out decision making and problem solving needs [21,73,82]

An additional shortcoming within Contributing Factors is that the concept Work/Environment Factors is primarily framed within an institutional model, neglecting to consider the blurred lines that exist within the homecare model. When care is moved into the home it changes the home environment into a care setting, thus blurring the lines as to where care ends and the home begins and vice versa. Patients and health care providers may have difficulty navigating this environment: providers consider the home their work environment, while patients consider the home their personal domain. However, maintenance, upkeep, and financial management of the home remain the responsibility of the patient. The literature shows that patients living alone, and thus bearing these responsibilities alone, run a higher risk of loneliness, anxiety, depression, and risk of non-adherence [19,34,40,53,56]. Additionally, these patients may have an increased need for follow-up care visits [19,34,40]. This demonstrates once more the importance of considering the physical environment beyond that of an institutional setting in order to adequately consider the dynamics of those living and working within the home.

It is apparent that while existing concepts within this class were representative of some of our findings, others were neglected, reflecting a lack of sensitivity and specificity to the homecare setting.

### Patient outcomes

Our last two safety markers *Out of pocket* and *My health for yours*: *declining caregiver health* mapped to the high-level class Patient Outcomes. ICPS defines this as the class *that contains the concepts that relate to the impact upon a patient which is wholly or partially attributable to an incident*. *Patient outcomes can be classified according to the type of harm*, *the degree of harm*, *and any social and*/*or economic impact*. There is a relationship between the descriptive classes, such as Patient Characteristics and Contributing Factors, and this high-level class.

*Out of pocket*: *the cost of caring at home* is the safety marker that refers to the emotional and economic burden that caring at home can create for patients and their caregivers. For instance, common economic and emotional aspects of receiving care at home include changes in financial status or well-being, stress, poor or uncertain quality of care, and depression [35,66,75]. The Patient Outcomes class includes the concept of Social and/or Economic Impact to which our safety marker *Out of pocket* maps well. For example, a common economic challenge for patients and caregivers (although caregivers are not considered in this class) is the loss of wages or reduction in wages because either the patient’s health limits his/her ability to work or the caregiver must reduce work hours in order to care for the patient [30,83,84]. Having to adjust to decreasing financial means, and sometimes lower-status employment through wage loss or reduction contributes to an increase in financial stress and a negative future economic outlook for both patients and caregivers [30,57,66,84,85].

In addition, the literature shows that patients and their families face personal expenses for needed home modifications, services, and equipment due to lack of financial coverage or difficulties in accessing funded support [30,39,48,86]. The economic and emotional impact on patients and caregivers strongly links to the status of their health and health outcomes.

*My health for yours*: *declining caregiver health* is a safety marker that describes how caregivers often sacrifice their own health for the health of their loved ones. Although caregivers are not included within the class of Patient Outcomes or its associated concepts, the literature shows a connection between the number and complexity of caring responsibilities, the caregivers’ health, and the health outcomes of the patient. For this reason, we have mapped this safety marker to Patient Outcomes and its concepts of Degree of Harm and Type of Harm. For instance, caregivers may experience depression, poor health, fatigue, exhaustion, and isolation which can affect the care they provide to their loved ones, resulting in safety risks and poor quality of care for the patients [30,57,66,75,85].

The types of harm facing family caregivers who provide care to patients with chronic illness include stress, fatigue, loss of sleep and concentration [33,35,39,59,61,67], as well as helplessness, anxiety, frustration, guilt, and strain [30,35,58,59,64,65,82,87],[88]. These problems can lead to varying degrees of harm from lower quality of life [61]; depression [76,82,89]; social isolation [18,29,87]; and psychological, physical, and emotional distress [18,30,39,61,79,90,91]; to, most significantly, increased morbidity [61] and mortality [61,63]. Hospital readmissions are consistently identified in the literature as patient health outcomes that result from caregivers’ poor health and diminished ability to care for patients safely [18,76,79,82,87,92,93].

It was possible to link these two safety markers, *Out of pocket* and *My health for yours*: *declining caregiver health*, to the class of Patient Outcomes, but as the health of the patient is inextricably linked to that of the caregiver [4] it would be far more prudent to include a high-level class that related directly to caregiver outcomes. Adapting the ICPS to consider the health of the caregiver and how it relates to the patient would add to the comprehensiveness of the classification system as well as patient health and safety. It has, for example, been shown that the level of emotional and mental health of the caregiver is positively linked to the psychological and emotional adjustment of the patient [61]. This interconnecting relationship should not be ignored and illustrates the need for an emphasis on patient/caregiver linkages within the ICPS concepts.

## Discussion

In our application of the WHO ICPS to our findings, we discovered that our seven safety markers mapped to three of the ten high-level classes. In working through these three classes and their associated concepts, we also discovered some lack of specificity relating to patient safety within the homecare setting (e.g. classes and concepts did not incorporate outcomes for unpaid caregivers), and that the clinical basis and structure of the ICPS also posed a challenge, particularly in applying findings from the scoping review to the system.

The clinical origins of the ICPS are apparent when examining the literature on patient safety associated with the WHO World Alliance for Patient Safety. For instance, their focus was on recognizing gaps in the knowledge regarding unsafe care and a high degree of error for *hospitalized* patients [94]. In order to establish priorities for research on patient safety, they established major topics in patient safety by examining *clinical* and organizational issues identified through types and causes of adverse events (AEs) that are harmful to patients [94]. When care is moved into the home it changes the home environment into a care setting, thus blurring the lines as to where care ends and the home begins and vice versa. The spirit of the classification system remains focused on institutional settings. This creates the potential to overlook the distinct and diverse needs and safety concerns located in the homecare setting, such as the provision of health care services, role of unpaid caregivers (friends/family members), and the characteristics and life circumstances of the patient (and caregiver) [95]. Furthermore, creating a classification system oriented toward a clinical institutional setting limits the applicability to the patient/caregiver perspective.

A second, and perhaps more important, point is the obscurity of the caregiver within the classification system. Caregivers are included within some of the ICPS concepts but failure to include the role of caregivers, and particularly caregiver outcomes, at the class level limits the specificity of the framework in capturing safety issues within the homecare setting. Homecare relies on the efforts and work of unpaid caregivers; if caregivers are ill or do not have the support they require, they cannot attend to the needs of the patient and may face their own declining health. This impacts the health and safety of both the patient and caregiver. It is integral to see the link between caregiver and patient health and safety; yet in the ICPS, the significance of caregivers has been reduced to one of incident reporting and being included within descriptions of incident characteristics. Concepts and classes specific to the homecare setting are required to maximize the applicability of the ICPS across all health care settings.

Finally, it was agreed, as others have reported [96] that a guide to using the ICPS within various settings would be beneficial. We also found to a degree, as Schulz et al. [13] observed, that there was limited guidance on how to deal with overlapping categories or classes.

## Conclusion

The scoping review findings resulted in the identification of markers relating to the safety of patients and caregivers. We did encounter some limitations in the application of the ICPS to homecare safety research. The ICPS, although applicable to our findings, did not effectively allow for locating all of the safety risks we identified within the homecare setting, due in part to a lack of specificity related to the patient caregiver linkages, and to a blurring of the lines that exist between the home and health care settings and the shared responsibilities that exist among patients, caregivers, and health care providers. In conclusion, although the intent in the development of the ICPS was to improve patient safety and care across disciplines and care settings, this will be achieved only by adapting the ICPS to reflect the uniqueness of the homecare setting. Following publication of this manuscript, we plan to engage with the WHO ICPS framework architects to discuss adaptations for relevance to homecare.

## Competing interests

The authors declare there are no financial and/or non-financial competing interests.

## Authors’ contributions

MM and AL contributed to every step in the generation of the evidence used to examine the usability of the WHO ICPS for safety in homecare, and shared the manuscript preparation; TB, JS, LS, KI and SD contributed to all aspects of the development of the knowledge used to examine the applicability of the ICPS to safety in homecare, and the review and editing of this article. TB and MM also incorporated the ICPS into the article and conducted the mapping of the safety markers. All authors read and approved the final manuscript.

## Pre-publication history

The pre-publication history for this paper can be accessed here:

http://www.biomedcentral.com/1472-6963/13/191/prepub
